# Laparoscopy to predict the result of primary cytoreductive surgery in advanced ovarian cancer patients (LapOvCa-trial): a multicentre randomized controlled study

**DOI:** 10.1186/1471-2407-12-31

**Published:** 2012-01-20

**Authors:** Marianne J Rutten, Katja N Gaarenstroom, Toon Van Gorp, Hannah S van Meurs, Henriette JG Arts, Patrick M Bossuyt, Henk G Ter Brugge, Ralph HM Hermans, Brent C Opmeer, Johanna MA Pijnenborg, Henk WR Schreuder, Eltjo MJ Schutter, Anje M Spijkerboer, Celesta WM Wensveen, Petra Zusterzeel, Ben Willem J Mol, Gemma G Kenter, Marrije R Buist

**Affiliations:** 1 Department of Obstetrics and Gynaecology, Academic Medical Centre, Amsterdam, The Netherlands; 2 Department of Obstetrics and Gynaecology, Leiden University Medical Center, Leiden, The Netherlands; 3 Department of Obstetrics and Gynaecology, Maastricht University Medical Center, GROW - School for Oncology and Developmental Biology, Maastricht, The Netherlands; 4 Department of Gynaecology, University Medical Center Groningen, Groningen, The Netherlands; 5 Department of Clinical Epidemiology Biostatistics and Bioinformatics, Academic Medical Centre, Amsterdam, The Netherlands; 6 Department of Obstetrics and Gynaecology, Isala Klinieken Zwolle, Zwolle, The Netherlands; 7 Department of Obstetrics and Gynaecology, Catharina Hospital Eindhoven, Eindhoven, The Netherlands; 8 Clinical Research Unit, Academic Medical Centre, Amsterdam, The Netherlands; 9 Department of Obstetrics and Gynaecology, TweeSteden hospital Tilburg, Tilburg, The Netherlands; 10 Department of Obstetrics and Gynaecology, University Medical Center Utrecht, Utrecht, The Netherlands; 11 Department of Obstetrics and Gynaecology, Medisch Spectrum Twente, Enschede, The Netherlands; 12 Department of Radiology, Academic Medical Centre Amsterdam, Amsterdam, The Netherlands; 13 Department of Obstetrics and Gynaecology, Erasmus Medical Centre, Rotterdam, The Netherlands; 14 Department of Obstetrics and Gynaecology, Radboud University Nijmegen Medical Centre, Nijmegen, The Netherlands

## Abstract

**Background:**

Standard treatment of advanced ovarian cancer is surgery and chemotherapy. The goal of surgery is to remove all macroscopic tumour, as the amount of residual tumour is the most important prognostic factor for survival. When removal off all tumour is considered not feasible, neoadjuvant chemotherapy (NACT) in combination with interval debulking surgery (IDS) is performed. Current methods of staging are not always accurate in predicting surgical outcome, since approximately 40% of patients will have more than 1 cm residual tumour after primary debulking surgery (PDS). In this study we aim to assess whether adding laparoscopy to the diagnostic work-up of patients suspected of advanced ovarian carcinoma may prevent unsuccessful primary debulking surgery for ovarian cancer.

**Methods:**

Multicentre randomized controlled trial, including all gynaecologic oncologic centres in the Netherlands and their affiliated hospitals. Patients are eligible when they are planned for PDS after conventional staging. Participants are randomized between direct PDS or additional diagnostic laparoscopy. Depending on the result of laparoscopy patients are treated by PDS within three weeks, followed by six courses of platinum based chemotherapy or with NACT and IDS 3-4 weeks after three courses of chemotherapy, followed by another three courses of chemotherapy. Primary outcome measure is the proportion of PDS's leaving more than one centimetre tumour residual in each arm. In total 200 patients will be randomized. Data will be analysed according to intention to treat.

**Discussion:**

Patients who have disease considered to be resectable to less than one centimetre should undergo PDS to improve prognosis. However, there is a need for better diagnostic procedures because the current number of debulking surgeries leaving more than one centimetre residual tumour is still high. Laparoscopy before starting treatment for ovarian cancer can be an additional diagnostic tool to predict the outcome of PDS. Despite the absence of strong evidence and despite the possible complications, laparoscopy is already implemented in many countries. We propose a randomized multicentre trial to provide evidence on the effectiveness of laparoscopy before primary surgery for advanced stage ovarian cancer patients.

**Trial registration:**

Netherlands Trial Register number NTR2644

## Background

Epithelial ovarian cancer (EOC) is the leading cause of death in gynaecologic malignancies. Ovarian cancer is usually diagnosed in an advanced stage, when tumour has spread from the ovaries throughout the abdominal cavity or into the liver parenchyma and pleural cavity (FIGO stage III or IV respectively). In advanced stage disease many patients have multiple tumour deposits spread out over the peritoneum, peritoneal carcinosis. Although survival of early stages is high, the survival of advanced stages is low. Despite an initial response rate of 80% after first line treatment, recurrences occur in 70% of patients, and the expected overall survival is 2 to 4 years.

Standard treatment of patients with advanced disease is primary cytoreductive (debulking) surgery (PDS), intended to remove all visible tumor localizations [1]. Surgery is followed by six courses of chemotherapy consisting of Paclitaxel and Carboplatin. Result of debulking surgery is the most important prognostic factor for survival [1-4] Leaving no residual tumour gives the best survival. However, if complete resection is not possible, the goal of surgery is to achieve at least residual disease smaller than one centimetre in diameter. The present rate of patients with residual tumour smaller than one centimetre in Europe is only 20-62% [5,6]. In case a tumour deposit of more than one centimetre is left at PDS some patients will be operated again after three courses of chemotherapy, a so called interval debulking surgery (IDS). Only those patients for who PDS was not considered to a maximal attempt by a gynaecological oncologist are candidate for this intervention [7]. A PDS leaving more than one centimetre of tumour is an unsuccessful laparotomy leading to more morbidity without gain in survival. It lengthens hospital stay and time of treatment and increases costs and should therefore be avoided.

Computed tomography (CT) is now used in pre-operative staging of patients with an ovarian tumour for predicting operability and to determine treatment [8-10]. CT criteria have been developed which are used to select patients for primary surgery [9]. Bristow et al. developed a model based on 13 criteria, like peritoneal thickening or bowel mesentery involvement, achieving an overall accuracy of 93% in predicting successful cytoreduction [11]. However, this result could not be achieved using the same criteria in another patient population [12]. Recently, Ferrandina developed a predictive score based on CT criteria as well as performance status [9]. Depending on the model and the predictive score used, 33% to 48% of patients would have had a suboptimal debulking, despite the prediction that complete removal would be feasible. Although CT is at present the most predictive procedure, it is not accurate enough to guide clinical management. [12,13]

Recently a randomized study of the European Organization of Research and Treatment of Cancer-Gynaecological Cancer Group (EORTC-GCG) and the NCIC-Clinical Trials Group comparing PDS and chemotherapy with neoadjuvant chemotherapy (NACT) followed by IDS was conducted [6]. Although survival was comparable in both groups, a subgroup analysis showed that patients with metastases with a diameter of less than five cm at start of primary debulking have a better prognosis when treated by PDS. Emphasizing the fact that PDS should be the standard treatment and that neoadjuvant chemotherapy should be reserved to patients in whom optimal debulking is deemed not feasible or who can not tolerate the procedure [6,14]. Therefore, selection of patients is very important and could be done by using laparoscopy to predict operability results [6,15].

Several prospective and retrospective studies have investigated the use of laparoscopy to predict outcome of debulking surgery. In a pilot study by Fagotti et al. [16] laparoscopy predicted debulking leaving tumour residual more than one centimetre in 100% of cases and debulking surgery with no macroscopic tumour left in 89% of cases [16]. With these data Fagotti et al. developed a prediction model with accuracy for prediction of unsuccessful debulking between 69% and 75% depending on the cutoff level of the Predictive Index Value used [17]. However, validation of this prediction model in another study population by Brun et al. showed that 56% of patients who were thought to have debulking until less than 1 cm of tumour residual underwent a unsuccessful resection [18].

Despite the absence of strong evidence and despite possible complications laparoscopy is already implemented in many countries. In this respect, we propose a randomized controlled clinical trial in which the outcome of PDS after diagnostic laparoscopy is compared with the outcome of PDS after standard diagnostic work-up.

## Objective

The aim of this study is to asses whether diagnostic laparoscopy can prevent unsuccessful debulking surgery in patients with advanced ovarian cancer. This study will also evaluate whether adding laparoscopy is cost-effective, improves quality of life and generates less morbidity in this population as compared to standard diagnostic work-up.

## Methods/Design

### Trial Design

**Figure 1:**
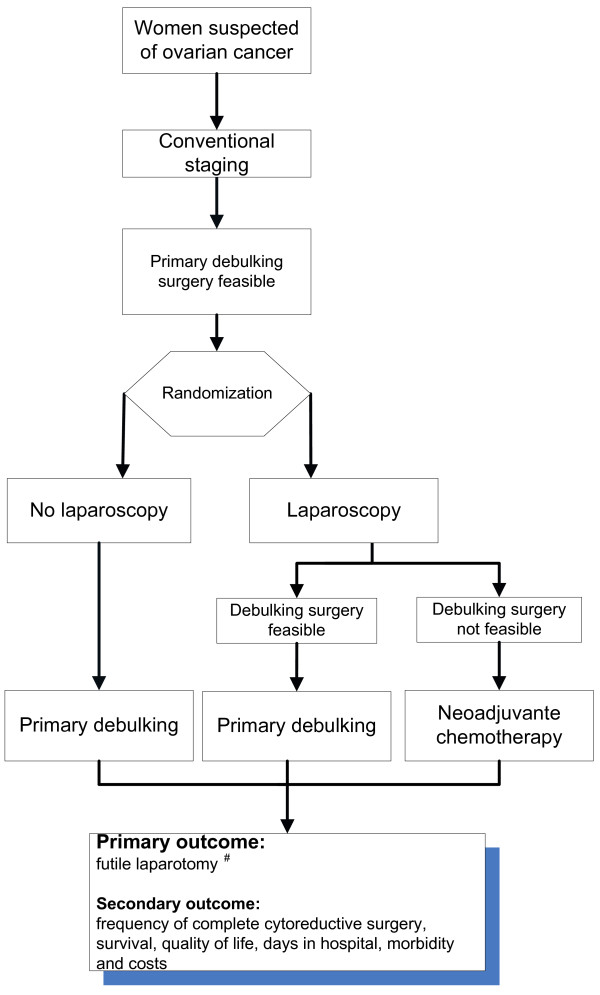
**LapOvCa-trial design**. * = Physical examination, ultrasound, tumor markers, chest X-ray, abdominal CT. ^# ^= Largest residual tumor localization, left behind at the end of cytoreductive surgery, is more than 1 cm in diameter. •Debulking surgery feasible: residual tumor after surgery will be < 1 cm. •Debulking surgery not feasible: residual tumor after surgery will be > 1 cm.

This study is a multicentre prospective randomized controlled trial in which all nine Dutch gynaecological-oncology centres and affiliated hospitals are participating. Patients with advanced ovarian cancer planned for PDS will be randomized to PDS or an additional diagnostic laparoscopy followed by either PDS or NACT and IDS. The rate of futile PDS in both groups (Figure 1) will be compared. A debulking surgery is regarded futile when the diameter of the largest residual tumour deposition is larger than 1 cm in diameter. The study is conducted according to the principles of the Declaration of Helsinki and in accordance with the Medical Research Involving Human Subjects Act (WMO) and has been approved by the ethics committee of the Academic Medical Centre Amsterdam (ref. no MEC 10/183). The protocol is registered in the Netherlands trial register number NTR 2644

### Eligibility criteria

#### Inclusion criteria patients

Patients with advanced ovarian cancer who are planned for primary debulking surgery are eligible for this study. Patients have to be able to give written informed consent and aged between 18 and 80 years.

#### Exclusion criteria patients

Contraindications for PDS are exclusion criteria for this study. These include a WHO performance status of ≥ 3 and a large immobile pelvic tumour giving the impression that complete debulking is not feasible. Further exclusion criteria are imaging techniques suggestive of intrahepatic metastatic disease of > one centimetre, extra-abdominal metastatic disease (excl inguinal lymph nodes or pleural fluid), peri-aortic lymphadenopathy larger than one centimetre above the level of the renal veins, extensive peritoneal carcinomatosis at the level of the diaphragm giving the impression that surgery leaving no macroscopic tumour residual is impossible and extensive bowel mesentery involvement.

### Patient recruitment, randomization and collection of data

Eligible patients are identified and counseled by the gynaecological staff of participating hospitals. Patients suspected of having advanced ovarian cancer are usually referred to a general gynaecologist. According to the national guideline for ovarian cancer, appropriate treatment as well as timing and localization of surgery will be discussed with a gynaecological oncologist from a centre hospital. All patients will undergo conventional staging, consisting of medical history, complete physical and gynaecological examination, ultrasound examination, assessment of CA 125 and CEA serum levels, chest X-ray and contrast enhanced abdominopelvic CT. CT scanning will be performed using the standard equipment in the hospital in which the patients will undergo laparoscopy. All CT's will be evaluated by an experienced radiologist and will be reviewed by the centre radiologist.

The decision that a patient is eligible for PDS will be made by the gynaecological oncologist in collaboration with the referring gynaecologist on the basis of all available information. All patients considered to be optimally operable will be offered PDS and will be asked to participate in this study. After written informed consent has been obtained, randomization will take place.

Randomization is performed by accessing a central internet-based randomization program and is stratified by gynaecologic-oncologic centre hospital. Patients will be randomly allocated in a 1:1 ratio to two groups. The first group will undergo PDS followed by chemotherapy and the second group will undergo an additional diagnostic laparoscopy. At study entry baseline demographic characteristics, medical history and findings of conventional staging are recorded in a case record form (CRF). After randomization, but before surgery, patients are asked to fill out a questionnaire consisting of items regarding quality of life (EORTC QoL-C30 and QoL-OV28, EQ-5D) and a questionnaire on additional homecare (SF-HLQ). The same questionnaires are also asked to be completed three months after start of treatment and six weeks upon ending treatment. At local centres, data collection is the responsibility of the local participating gynaecologist and research nurse. The data collected in this study are coded and processed with adequate precautions to ensure patients confidentiality.

### Interventions

Debulking surgery should take place within 6 weeks after randomization. When patients receive a laparoscopy this should be done within 3 weeks after randomization. After the laparoscopy the decision is made for PDS or NACT with IDS after three courses of chemotherapy. The diagnostic laparoscopy will, when possible, be performed by the referring gynaecologist in attendance of the gynaecological oncologist. In case the laparoscopy can not be attended by the gynaecological oncologist and the laparoscopy can not be performed in the centre hospital, the laparoscopic procedure will be recorded completely. An open laparoscopy has to be performed examining systematically the ovaries, fallopian tubes, uterus, pelvic peritoneum, omentum, serosa and mesentery of the large and small bowel, spleen, liver surface, paracolic gutters and diaphragm. Tumour localizations will be documented in size and position to adjacent structures. To confirm diagnosis of ovarian cancer biopsies of tumor localizations will be taken. Judgment for incomplete cytoreduction will be made by the gynaecological oncologist on the following parameters:

- Extensive agglutinated intra-abdominal metastatic disease

- Extensive serosal invasion of the intestines making multiple bowel resections or more than 1,5 m of bowel resection necessary in order to reach complete cytoreductive surgery.

- Extensive peritoneal carcinomatosis at the diaphragmatic level

After laparoscopy patients will be submitted to either PDS within three weeks, followed by six courses of chemotherapy consisting of Paclitaxel and Carboplatin or to NACT followed by IDS and subsequent adjuvant chemotherapy. Both primary and interval debulking surgeries will be performed in the center hospital or in the referral hospital in collaboration with a gynaecological oncologist according to standard procedures for advanced ovarian cancer. The surgeon will describe the localization and diameter of all tumor depositions before surgery and of residual tumour in the surgical report upon ending surgery. This will be done for 11 abdominal regions. The amount of metastases will be classified as 0, 1, 2-10, 11-50 and > 50 tumour deposits. The size of the largest metastasis will be categorized into 0 mm, ≤ 10 mm, 11-20 mm, 21-50 mm, 51-100 mm and ≥ 100 mm in diameter. Furthermore the surgical procedures, total length of the operation and blood loss will be recorded.

### Follow-up

As the primary outcome measure is the rate of suboptimal debulking surgery, this will be assessed at the end of the surgical intervention. Data for secondary outcomes will be assessed peri-operatively, during treatment and at routine follow-up. All data will be registered on a case record form (CRF) by the treating physician and checked by the research nurse (status review). For the economic evaluation use of health care resources is assessed as part of the clinical data collection (CRF) and additional patient questionnaires. The doctor registers resource utilization on a CRF related to the use of operation time, duration of hospital ward and ICU stay, additional diagnostic or therapeutic interventions. The questionnaire addresses health related resource use during follow-up, including visits to general practitioners and other primary care providers, outpatient visits and readmission, home care and informal care. Adverse events will be followed until they have abated, or until a stable situation has been reached. Data regarding quality of life (QoL-C30, QoL-OV28, EQ-5D,) are assessed with help of self reported questionnaires before start of treatment, at 3 months and at the end of treatment.

### Outcome measures

Our primary outcome measure is the proportion of debulking laparotomies with a largest residual tumour of more than one centimetre in diameter (futile laparotomy).

Our secondary outcome measures will be progression-free and overall survival, the number of debulking surgery leaving no residual tumour, debulking surgery in which the largest residual tumour is less than 1 cm in diameter, costs and quality of life.

### Statistical analysis

#### Sample size

Considering the present rate of debulking surgeries for ovarian cancer leaving more than one centimetre of residual tumour in the Netherlands after conventional staging is to be at most 40%, we estimate that after laparoscopy this should be less than 20%. With a two-sided significance level of 0.05 90 patients per arm have to be included to achieve a power of 80%. Considering 10% loss to follow-up and protocol violation, we plan to enroll 200 patients.

#### Data analysis

The results of the randomized trial will be analyzed according to the intention to treat principle. Difference in the proportion of futile laparotomies in both arms will be tested using a Chi-square test with a two-sided significance level of 0.05. Prognostic value of standard staging and staging with laparoscopy will be expressed as sensitivity, specificity and predictive values. Standard staging and standard staging with laparoscopy is considered true positive when subsequent suboptimal debulking surgery is correctly identified. Overall survival (OS) and progression free survival (PFS) will be measured from the date of randomization to respectively the date of death and first documented date of progression. OS and PFS will be calculated in both arms by the Kaplan-Meier method. Treatment complications will be reported in a contingency table. Quality of life will be compared between both treatment arms at various time points. We will make a subgroup analysis for number of futile laparotomy for each gynaecologic-oncologic centre, FIGO stage and size of metastatic tumour seen at laparoscopy or laparotomy.

### Economic evaluation

The aim of the economic evaluation is to assess whether the laparoscopy can reduce the number of futile primary laparotomies and associated costs to an extent that at least offsets the costs of this laparoscopy in all eligible patients. A strategy that reduces the number of unnecessary laparotomies is considered preferable, even if this does not improve survival, also if the costs generated by both strategies are comparable. The economic analyses will be conducted from a societal perspective including direct medical and direct non-medical costs. Relevant costs components that will be taken into account are costs of the laparoscopy and laparotomy, operation time, hospital days, interventions for complications and intensive care admission. Indirect costs are associated with home care, consisting of both professional care as well as informal care.

## Discussion

Primary debulking surgery with the aim to leave no residual tumour is still considered as the standard treatment for patients with advanced ovarian cancer. However, more and more patients are submitted to IDS to reassure better surgical results. A complete debulking, without any residual tumour after surgery, should be pursued to obtain the best prognosis. However, the number of primary surgeries leaving residual tumor of more than 1 cm in diameter, is up to 40%, while during interval debulking surgery more often complete resection is achieved, without affecting survival [19,20]

Selecting patients who benefit from primary debulking surgery, i.e. in whom complete surgery results are possible, should be optimized. In several institutes diagnostic laparoscopy is already standard of care to determine further treatment in ovarian cancer patients. Prospective and retrospective studies showed that predictability of surgical outcome with laparoscopy is better than that of standard diagnostic staging [16-18,21]. However, prediction models could not be validated in different populations. Furthermore, laparoscopy is an invasive procedure under general anesthesia with a serious morbidity rate of 1‰-5% [22,23]. If laparoscopy before starting treatment is a reliable additional diagnostic tool in predicting result of PDS, unsuccessful laparotomies can be prevented. This will optimize treatment for the individual patient. To this date no randomized controlled trials have investigated whether additional diagnostic laparoscopy prevents unsuccessful laparotomies.

## Competing interests

The authors declare that they have no competing interests.

## Authors' contributions

MRB, GGK, PB, BO, BWM and MJR were involved in conception and design of the study. MJR and MRB drafted the first manuscript. All authors edited the manuscript and read and approved the final draft.

## Pre-publication history

The pre-publication history for this paper can be accessed here:

http://www.biomedcentral.com/1471-2407/12/31/prepub
